# Gleanings from Our Exchanges

**Published:** 1872-09-15

**Authors:** 


### Gleanings from Our Exchanges.

### OIL TURPENTINE IN HEMORRHAGE FROM THE BOWELS.

Dr. S. Wood, of Clyde, New York, in an article in the Buffalo *Medical and Surgical Journal*, August, 1872, advocates the use of oil of turpentine alone in large doses for the control of hemorrhage from the bowels, occurring as a complication of typhoid fever.

The following case is one among a number given in illustration:

*Case* 1.—In the evening of the second day of the month of September, 1852, I was requested to visit W——, a boy of sixteen years, with typhoid fever, some two and a half miles distant, and who had been under the care of another practitioner some two or more weeks. I was told that the case was one of great urgency, since an unfavorable prognosis had been given. On arriving at the bedside, I was informed that blood in large quantities was passing from his bowels at each frequent evacuation. Found patient exceedingly restless from pain and tympanic distension of the bowels. Skin dry and burning; pulse extremely rapid and thready; tongue dry, clean, and with dark papilla, with sordes on the teeth and lips. The prognosis indeed seemed most unfavorable.

In being called upon to prescribe in an emergency of this kind, there was an imperative demand for immediate and decisive action. What was to be done should be done quickly. Not a moment was to be lost. In running rapidly through my mind the various styptic remedies of the *materia medica*, suitable to the case before me, I happened to recollect an article first published in the *Medical Times*, August 17, 1850, from the pen of Dr. Wm. Budd, physician to the Bristol Infirmary, on the "styptic properties of oil of turpentine in a case of purpura hemorrhagica," and also another article in *Braithwaite's Retrospect* for January, 1851, by John Griffith, Esq., Wexham, on the use of turpentine in large doses, in uterine hemorrhage; and from the high praise given this remedy by these medical gentlemen, I at once resolved to give it a trial. Some was procured from a near neighbor, and, without delay, I administered a teaspoonful in some sugar and water, and in fifteen minutes as much more. After the expiration of an hour I gave half the quantity in the same manner, and then ordered that in two hours twenty drops should be given, and so on every two hours until I should see the patient again the following morning.

Sept. 3.—Symptoms much improved; pulse slower and fuller; less heat of surface, with a tendency to perspiration; expression of countenance less anxious; and, from the character of the stools, was fully convinced that the turpentine had controlled the hemorrhage almost immediately after the first dose had been taken. For the next twenty-four hours I ordered the remedy to be given in twenty-drop doses every four hours.

Sept. 4.—Patient still improving; symptoms all better; no more hemorrhage; but the turpentine was so obnoxious that I reluctantly discontinued its further use until I should see him again, and substituted a tonic in its stead.

Sept. 5.—Hemorrhage had returned with symptoms of a very threatening character. I now prescribed the turpentine again, to be given in twenty-drop doses, as first, every two hours for three or four doses, depending upon symptoms, and then every four hours until I should see him next day.

Without extending the report of this case further, I will briefly state that I continued the remedy some three or four days. At the expiration of this time convalescence was fully established, and without further drawback went on rapidly to complete recovery, there being no more hemorrhage.

In conclusion the author says:

To enter fully upon the therapeutics of the oil of turpentine, or history of its introduction into the materia medica, is not the object I have in view at this time. It would be too much of a trespass upon your time and patience, my sole purpose being to throw what light I can upon, or call your attention more especially to, the management of hemorrhage of the bowels, when it occurs as a complication in the latter stage of typhoid fever. It will be noted that the doses given are much larger than recommended by our works on materia medica in passive hemorrhages from the bowels. How the remedy acts, whether as a stimulant or styptic to an ulcerated surface, you can judge as well as myself. I am well aware that oil of turpentine has been much used of late years in the ordinary dose in certain stages, or to combat certain symptoms of typhoid fever, but not in the hemorrhage occurring during their progress.

Case of Excessive Hypodermic Use of Morphia.—Dr. Judson B. Andrews, Assistant Physician to the New York State Lunatic Asylum, Utica (*Am. Jour. Insanity*, July, 1872), mentions the case of a woman, aged 30 years, who had passed into an acutely maniacal condition, and exhibited scars and ecchymosed spots, covering nearly the whole of the body, which could be reached by her own hand. When convalescent, she asserted that she had employed the hypodermic injections for three and one-half years, once, and much of the time twice a day, making in all about two thousand injections; that during the last few months of its continuance she had used a drachm and one-half of morphia per week; that she inserted the needle perpendicularly to the surface, and often carried it its full length into the tissues. About four months before death the patient, in rubbing her hand over the breast, discovered an elevated point, just under the skin, which on pressure gave a pricking sensation. This was cut down upon and a broken needle extracted. From this time from one to twelve needles were removed daily from various parts of the body; from the left breast, the abdominal parietes, the mons Veneris, the labia and vagina, the thighs, from the leg down to the ankle, from the buttocks, from about the anus, from the back as high up as between the shoulders. 286 needles were taken from her body during life; 11 were found in the tissues after death; 3 were passed from the rectum during sickness; making a total of 300 needles and pieces. Of this number 246 were whole, and 54 were parts of needles. As regards position in the body, they were distributed about as follows: In right breast, 150; left breast, 20; abdomen, 60; genitals, 20; thigh and legs, 30; back, 20.

It was supposed that they were introduced through the skin while she was under the influence of morphia, hypodermically administered, and while suffering from hysteria. That some were found in positions where they could not have been inserted by the patient, can be accounted for by their movements in the tissue, which were observed so often during the life of the patient. Her mother could throw no light on the subject, but recalled the circumstance that the patient purchased, at one time, ten papers of needles, and could account for only two of them. They were not obtained or introduced while in the asylum.—*Med. Rec.*

University of Zurich.—The professors of this university are unanimously opposed to the admission of further female students, and they intend to apply to the federal government of Switzerland for a bill restricting the rights of studentship to males.—*Ibid.*

Pharyngitis and Rhinoscopy.—Harrison M. D., Surgeon to Philadelphia Hospital (*Philadelphia Medical Times*, Aug. 1, 1872), says that in pharyngitis, dependent upon general naso-pharyngitis, no instrument can approach in efficacy the atomizer. The best form of this instrument with which he is familiar is that known as the Sass sprayer. The peculiarity of this instrument consists in the test-tube receiver, which is held in the left hand, and a pair of very long barrels, the points of which, when the receiver is near the mouth, are lodged within the axis of the pharynx; the whole being worked by a bulb and tubing held in the right hand. In specific ulceration of the naso-pharyngeal space, he has obtained good results from the use of a solution of sulphurous acid of one drachm to the ounce, sprayed upward through the naso-pharyngeal aperture; or the pure acid may be applied to the affected spot if the part thus operated upon lie below the palate. Where there is abundant mucus, as in lingering acute catarrh, a spray of strong alum water proves oftentimes efficacious. It is in this class of cases that insufflations of alum are of advantage. The best insufflator is a simple glass tube, bent at convenient angles, and furnished with a fenestra at about its middle; a light piece of India-rubber tubing attached to one end of the glass tube completes the instrument. The powder to be used is inserted in the glass tube through the fenestra, which is then covered by a sliding cylinder of rubber. The instrument now being inserted in the pharynx, with the orifice of the tube pointing upward, the opposite end of the instrument is held between the lips of the operator, who quickly blows the powder up into the naso-pharyngeal space.

In closing this "Clinical Lecture," he advocates the nasal douche as an adjunct to the treatment; more, however, as an aid in *washing* the parts than to medicate the region. Weak solutions of salt, or carbonate of soda, used *tepid*, will meet every indication. The washing need not be repeated oftener than once a day—say at the time of the morning toilet.—*Med. Rec.*

Hydrochlorate of Ammonia.—Spencer Thomson, M. D., Ashton, Torquay (*British Medical Journal*, July 13, 1872), finds the hydrochlorate of ammonia one of the most efficient remedies in cases of portal dropsy, prescribed in scruple or half-drachm doses, given tolerably diluted, every six or eight hours. The recent accounts of the success attending the employment of this remedy in hepatic affections in India, he hopes will lead to its more extensive use in England.

Foreign Appointments and Changes.—Dr. Ferhart, of Jena, has been appointed to fill the chair of Clinical Medicine in Wuerzburg, vacated by Bamberger, who has gone to Vienna; and Dr. O. Liebreich (discoverer of the action of chloral) has been appointed Ordinary Professor of Materia Medica in the University of Berlin. Dr. Brown-Sequard has resigned the Chair of Comparative and Experimental Pathology in connection with the Faculty of Medicine of Paris, and M. Vulpian has applied for the position. The College of Professors of the University of Vienna have determined on adding a third teacher of Clinical Midwifery to the two already existing, and have appointed to the new post Dr. Gustav Braun, Professor of Midwifery in the Joseph's Academy. Mr. Hancock, for many years Surgeon to Charing Cross Hospital, has been appointed to the Presidential Chair of the Royal College of Surgeons.

Dr. P. A. Simpson has been elected Professor of Medical Jurisprudence in the University of Glasgow.—*Med. Rec.*

Aphonia.—Meyer, of Berlin, reports the case of a young girl with a crowing cough, the sequel of whooping-cough, which lasted from two to three minutes, and was repeated every quarter or half hour. The paroxysms terminated in aphonia. The disease increased in spite of the most active treatment. After two years, electric medication was resorted to. Meyer stimulated the nerve of the larynx by placing one of the electrodes in the direction of the recurrent laryngeal nerve, on the internal edge of the sterno-cleidomastoid, in the groove between it and the tracheal artery and œsophagus. The inferior electrode was placed on the transverse process of one of the cervical vertebræ, in order to be as near as possible to the origin of the vagus nerve. After sixteen seances the attacks had already intermissions of from two to three hours, and at the end of twenty other seances they ceased entirely, and with them the aphonia disappeared.—*Am. Jour. Obstetrics.*

Good Effects of Aconite in Acute Pneumonia.—Among other cases of interest which came under the observation of Dr. Murchison, in the male wards of St. Thomas' Hospital, London, was that of a boy aged fifteen, who was admitted with acute pleuro-pneumonia of the right side, and herpes labialis, and considerable increase of temperature. On the administration of eight minim doses of tincture of aconite, with liquor ammoniæ acetatis every four hours, the temperature at once came down, and the disease did not increase.—*British Medical Journal*, July 13, 1872.

Cyanosis from Nitrate of Silver Removed by Iodide of Potassium.—Dr. L. P. Yandell, jr., Professor of Materia Medica and of Clinical Medicine in the University of Louisville, says, in the *American Practitioner :*

Most practitioners have met with cases of cyanosis produced by nitrate of silver; and such cases were more frequent many years ago, when nitrate of silver was more frequently employed for epilepsy than it is at the present time. According to most authorities the stain is permanent, and not amenable to treatment. Many remedies have been suggested, iodine, nitric acid, and acid nitrate of potash being the favorites; but I have found no cure recorded. As much as fifteen grains of nitrate of silver have been given thrice daily, in pillular form, without injury; but five grains in solution seems to be the largest dose capable of safe administration. It is the generally accepted opinion that the blue skin never supervenes when the remedy is given for a less period than three months. The discoloration first begins about the gums and fauces. It has been found in the coats of the intestines and eyes. It may appear several months after cessation of the use of the medicine; and exposure to the sun seems to predispose to its development.

The stain has been variously described as blackish, bluish, grayish, slate color, and bronze. The mineral is deposited in the deeper parts of the skin, and is most abundant where the skin is most vascular. A blister upon the skin produces a white vesicle, as in the normal cuticle.

The two cases which have suggested this report, are similar in many respects. Both were young merchants, and both had been treated unsuccessfully for epilepsy by nitrate of silver in their youth. Both contracted syphilis, and for tertiary symptoms got iodide of potassium. This drug was given in from ten to sixty grain doses, thrice daily, for a number of months, in connection with ferruginous or bitter tonics. One of the patients was forced to discontinue the iodide because of its disagreeable effect upon the system. The other took it until all traces of syphilis had passed away, and he increased in flesh under its use. In both cases the fading of the stains was gradual. In the first case there is a faint trace of discoloration remaining, though it is scarcely perceptible. In the second, which was much the darker of the two, there is not a shadow of the disfigurement. The iodide of potassium was not given in either case with reference to the cyanosis, and its beneficial effects were observed by me accidentally more than a year after their occurrence. It may be well to state that both patients were treated by the moist mercurial vapor bath during much of the time that they were using the iodide of potassium, and the abundant diaphoresis may have assisted the action of the iodide. I would suggest, therefore, for the treatment of nitrate of silver cyanopathia, the use of the vapor bath in connection with the iodide of potassium.—*Canada Medical Record.*

Ammonia in Suspended Animation.—The value of the injection of ammonia, as recommended by Prof. Halford, in cases of snake-bite and suspended animation, has been again demonstrated. A lady in Melbourne recently swallowed by accident an ounce of Browne's chlorodyne, which is a mixture of chloroform, morphia, and prussic acid. When seen by her medical attendant, she was, as he imagined, on the point of death—cold, insensible to everything, and giving only occasional gasps as signs of breathing. Recollecting a former case, in which a young man who had taken chloroform was revived after death had apparently occurred, the doctor mixed half a drachm of the liq. ammon. fort. with one and a half of water, and within the space of one minute injected the whole into a vein of the arm. In a few minutes the pulse returned, the breathing became natural, and by twenty minutes the whole body had regained its natural warmth ; but perfect consciousness did not return for some hours afterward. The patient made a rapid recovery. Two further instances have also been reported in which the timely use of the injection saved the victims of snake-bite from the death which threatened them.—*Melbourne Argus.*

Grafting with Rabbit Skin.—There have been reported to the Paris Academy of Sciences three cases of ulcers in the human subject healed by grafting upon them cutaneous particles taken from the rabbit. M. Larrey was so pleased with the idea that he proposed entering the Report for a prize. Whether the new surface produced a growth of rabbit's fur, we are not informed. But why not ? And if so, why not put the new practice to profit by substituting for the rabbit the Angora goat or some other variety which will produce a valuable wool ? Baron Munchausen did something of this kind, if our memory serves us, with his horse. There are some thousands of vagabonds and convicts in California, who might be made valuable to the State, or at least who would pay for their keeping, if, by the aid of delicate surgery, they were set to raising wool.

Influence of Rheumatism on Character.—In a translation of Dr. Faure's article on this subject (*Archives Generales*, Sept. 14, 1871), by Dr. S. G. Webber, of Boston, Mass., in the *Journ. Psychological Medicine*, it is shown that rheumatism is manifested under such variable forms that one may inquire, on meeting anything unusual in a patient subject to its attacks, whether rheumatism may not be concerned therein. Why may it not attack the organs of the cerebral functions on which character depends, as well as the heart, etc.?

A man who is subject to rheumatism will very often state that he has moments of despondency without cause, of inquietude, of forlornness, inexplicable to himself. Then he is discouraged without cause, and sees every thing in the shade ; that which ought scarcely to be the object of a slight care becomes the cause of a cruel torment. He is without force, his thoughts can be fixed on nothing, all intellectual work is impossible. If he wishes to solve a problem he soon experiences fatigue and heaviness of his head, which often turns into a violent headache. Then his sensations are altered, his affections cease, he is indifferent to everything. That which has the most right or power over his mind, remembrances which are most dear or most painful, have no interest for him. His character has changed. He is conscious of his condition, and can for a few minutes rouse himself out of it. A crisis may follow, his head is congested, he feels quite giddy. Finally all these symptoms disappear, and his mind recovers its tone and clearness. The attacks vary much with individual disposition.—*Med. Record.*

Epidemic of Jaundice.—An epidemic of icterus prevailed in Paris during the past winter. It is described by Dr. Decaisne, before the Academy of Medicine, as attacking persons in all conditions of life and health, without any assignable general or local cause. The *velum* of the palate exhibited the general yellowness. A strict diet, with the free use of cream of tartar lemonade, was sufficient in general to effect a cure. The physicians of the Academy appear to have regarded the epidemic as without a precedent. In the *Medical Press* for February, 1866, will be found a record made by the senior editor of this journal, of a similar epidemic which prevailed extensively in Vermont in the autumn of 1865, and which attacked nearly one-half the population of some towns. In that instance the mercurial treatment was found successful and necessary.—*Pacific Medical and Surgical Journal.*

The Work done by a Human Heart.—The total daily work done by the human heart is equivalent to 124,208 tons lifted one foot. The daily labor of a workman, deduced from long-continued observations of various kinds of labor, is found to be equal to 3,540 tons lifted through one foot during the ten hours. This is less than three times the work done by a single heart, beating day and night for twenty-four hours. In a boat race, it is calculated that 15 foot-pounds of work are performed by each ounce of muscle during each minute of the rowing. No muscular labor that man can undertake is more severe than this; and yet this labor is only three-fourths of that which is exerted day and night during life by each of our hearts. If the heart should expend its entire force in lifting its own weight vertically, it could raise that weight 19,754 feet in an hour. An active pedestrian can climb from Zermatt to the top of Monte Rosa, 9,000 feet, in an hour ; or he can lift his own body at the rate of 1,000 feet an hour, which is only one-twentieth part the energy of the heart. The heart's energy equals one-third of the total daily force of the muscles of a strong man. It exceeds by one-third the labor of the muscles in a boat race, estimated by equal weights of muscle ; and it is twenty times the force of all the muscles used in climbing, and eight times the force of the most powerful engine of the same size yet invented by man.—*Boston Journal of Chemistry.*

Means of Arresting Epistaxis.—Dr. Martin, of Geneva (*Journal de Med. et de Chir. Prat.*), says that, as the blood in general in epistaxis comes only from one nostril, and most frequently from the anterior third of one of the nasal fossæ, he compresses the facial artery of the side upon the upper jaw, very near the nose, and thus diminishes the effusion of blood into the nasal cavity, which almost always arrests the epistaxis. He has had the pleasure of stopping very obstinate bleedings from the nostrils in the streets, on steamboats, and in coaches, or in the theater in this way. In the schools of his Canton some professors have recourse to it with the boys, and have found it to answer well. He adds that in two cases this plan ceased; but in three cases plugging of the nostrils also failed. Patients were drunkards.

New York Physicians.—According to the New York *Medical Register* for 1872, there are in New York city, Brooklyn, and vicinity, 1,748 practising physicians in good standing, a gain of 195 over the year 1871.—*Med. Rec.*

CURE OF STRANGULATED HERNIA BY ASPIRATION.—M. Demarquay communicates to *Le Mouvement Medical* a case of successful reduction of, and recovery from, strangulated congenital inguinal hernia by means of aspiration. A young man, 31 years of age, after fatigue during the 5th of May, while on a visit at Vermillion, was suddenly seized with colic, attended by frequent vomiting; a tumor of considerable size at the same time had formed in the left groin. On the following day these symptoms continuing, he was taken to the surgical *servir* of the *Maison de Sante*, where the *interne*, having unsuccessfully employed the taxis, applied a bladder filled with ice, and waited for the morrow. On the morning of the 7th the patient was seen by M. Demarquay. He was then feverish and thirsty; the tumor voluminous, and it was seen that its precise nature was that of strangulated congenital hernia, of which M. Demarquay states he had never previously successfully treated a case by operation. The employment of the taxis having failed, the patient was put under the influence of an anæsthetic, and again the same measure taken with no better result. It was then determined to employ aspiration. A fine trocar was inserted at the center of the tumor, and by means of the aspirator of M. Potain 120 grammes of fluid were extracted from the intestine, without counting the gas. The tumor instantly collapsed; the trocar was removed; an interval of a few minutes allowed to elapse before further interference took place. No swelling took place, nor was there indication that further escape of gas or liquid from the intestine was going on; the taxis was again very gently applied, pressure was made very gently above and below the tumor, and the intestine felt to glide back into the cavity of the abdomen. The patient was subsequently treated by perfect quiet, with low diet, opium in small doses being given, and, beyond the occurrence of some degree of inflammation in the testicles from the pressure and handling it had undergone, no accident took place.—*The Doctor.*

AMERICAN MEDICAL ASSOCIATION. — The Triennial List of Permanent Members will be published this year. Permanent members who have not paid their assessment will please notice :

"Any permanent member who shall fail to pay his annual dues for *three successive years*, unless absent from the country, shall be dropped from the roll of permanent members."

WM. B. ATKINSON,
*Permanent Secretary.*

THE CENSUS.—NOT ENOUGH GIRLS TO GO ROUND.—It is reported that the complete census returns of the United States for 1870 give us these startling statistics of our population :

| | |
|---|---|
| Men and boys | 19,493,665 |
| Women and girls | 19,064,806 |
| Surplus of men and boys | 428,859 |

A surplus of 428,859 men and boys is something really startling, when it is considered that Adam and Eve, one man and one woman, is the law of the creation. But as in the chapter of accidents there are more fatalities among boys than girls, and more widows than widowers, and more old maids than old bachelors, our surplus of men and boys is in the infantile and not in the adult population. "So time at last makes all things even."—*Medical Record.*

EXAMINATION OF MIDWIVES.—In order to repress some of the dangerous abuses caused by want of examination, and consequently of education among "monthly nurses," the Obstetrical Society of London has resolved to form a jury from its members, and to deliver certificates to those women who, on examination, shall show themselves to be capable of executing certain obstetrical manipulations, and who shall show that they have had sufficient elementary instruction.—*Ibid.*

OPIUM EATING.—The Legislature of Kentucky, in order to check the practice of opium eating, which is greatly on the increase, has just passed a bill that, on affidavit of two respectable citizens, any person who, through excessive use of opium, arsenic, hashish, or any drug, has become incompetent to manage himself or his estate, may be confined in any asylum, and placed under guardianship, as in the case of habitual drunkards or lunatics.—*Ibid.*

COTTON SEED OIL.—There are at present upwards of twenty mills in this country exclusively operated in the manufacture of oil from cotton seed; and over 150,000 tons of seed are used annually. The oil cake is sent largely to England, where it is used as food for cattle. The oil goes mostly to Bordeaux, Barcelona, and other olive-growing sections of Europe, from whence, after "doctoring," it comes back as "pure olive oil."—*Ibid.*

LEGALIZED DISSECTIONS. — Michigan and Iowa have recently legalized dissections, and made provision for it by permitting physicians, medical societies, and colleges to claim for this purpose the bodies of criminals and paupers unclaimed by friends.

### PRESCRIPTIONS FOR PRESCRIBERS.

The following note, preceding a table of maximum doses, is taken from the *Pharmacopœa Helvetica*, 1872:

"Doses maximæ medicaminum heroicorum, quas dispensando transgredi non licet nisi medicus id expresse postulaverit numerum exprimendo verbis linea subnotatis, et signo (!) distinctis."

None but the initiated can form an adequate idea of the anxiety and vexation in the dispensing of medicines, which arise from causes apparently trivial, though in reality of serious nature. We may mention among these, indistinctness in the writing of the prescription, due to the use of the lead pencil; illegibility from careless chirography; the omission of directions for the use of the medicine, etc. The most grievous annoyance to the apothecary, however, is the general neglect, on the part of physicians, of the rule suggested in the above quotation; that is, the prescribing of heroic doses of powerful agents—doses which, under ordinary circumstances, could be given only with dangerous, if not fatal, results—with nothing to assure the dispenser that an error has not been committed. Now, mistakes in prescriptions have occurred, and will again occur, despite the care of the physician; and to shield the patient from the results of such possible errors is within the province of the apothecary. What, then, is his duty, upon receiving a prescription which contains either a palpable error or an extraordinary, anomalous dose? It is clear enough, and, to his credit be it said, it is generally put in practice. He must consult the physician before dispensing the prescription. Making, therefore, a plausible excuse for delay, and arranging, if possible, to send the medicine when "finished," he dispatches his trusted apprentice *sub rosa* (out the back door) with instructions to find the doctor and make the proper inquiries.

Since most of the errors of which we complain are the fruits of the hurry of a large business, it generally happens that the apothecary's Mercury does not immediately stumble upon the object of his search. So it often occurs that the impatient patient sits on the stool of expectancy hour after hour, awaiting the results of the prolonged labor of that "young man" who is "working at his prescription."

Now, the returning of a prescription to a doctor is naturally disagreeable to him. It bears with it at least a suspicion of error on his part. If there be no error, the doctor too often, without due appreciation of the caution of the apothecary, manifests by word or manner a disposition to resent the calling in question of his accuracy. If, on the contrary, the physician finds that he has made an error, he seldom takes occasion to thank the apothecary for his scrutiny or consideration. For the most part he acknowledges the mistake and makes the correction with a complacent smile, calculated to exculpate himself by a show of belittling the error, albeit this may have been as fatal to his patient as was the thrust of Tybalt to Mercutio.

To avoid these very unpleasant embarrassments and misunderstandings, prejudicial to the doctor, apothecary, and the patient, we urge the adoption by all physicians of the rule given in the *Pharmacopœa Helvetica*, to wit: that in prescribing unusual doses of poisonous medicines, the physician should, in addition to the common signs, *indicate the quantity by writing it out in full*, *underscoring* the words, *and adding* the sign (!). Thus:

℞ Morphiæ Sulphatis, gr. x. *ten grains* (!)

Divide into 6 (six) powders.

An observance of this rule by physicians is obligatory in several European states, and a similar requirement in our country would be of great advantage.

We cannot leave this subject without another suggestion upon the art of prescription writing, namely, the importance of attaching directions for the use of medicines. It is a wise precaution against mistake and misuse, and has manifold advantages. An example may serve in illustration. We lately saw a prescription for powders, each of which contained thirty grains of digitalis, with no directions as to their use. A very proper degree of caution on the part of the dispenser caused him to make inquiry of the prescriber, only to learn that the powders were to be made into a lotion for external application.

Another and more forcible instance we have in the following case, which occurred in our experience. A prescription calling for sul. morphia, gr. x, was presented. No directions to indicate its use accompanied it. Suspecting something wrong, the dispenser questioned the patient, and learned that he was suffering from ague, and had been told to take "half the powder to night, and half to-morrow morning." Of course quinæ sulp. was substituted, and the patient departed none the wiser of his near approach to death. Had this prescription appeared at a less particular establishment, the result might have been different; whereas, had the directions been given, they would have served as a safeguard against mistake in any store.

In conclusion, we will repeat the suggestions of the foregoing remarks:

*First.* Physicians should make an invariable practice of indicating heroic doses by repeating the quantity desired in written characters, underscoring the same, and attaching the sign (!).

*Second.* When ordering powerful medicines as local applications, they should so far indicate their use as to remove suspicion of error from the mind of the dispenser.—*Pharmacist.*

Vienna Obstetrical Hospital.—In the three different departments of this hospital there are annually from 8,000 to 9,000 deliveries. In Prof. Braun's clinic, during 7¾ years, from 1863 to 1871, among 34,851 births, 363 died of puerperal peritonitis, including all cases operated upon, *i. e.*, 1.04 per cent., or 10 of every 1,000 deliveries; and 111 died of accidental diseases, *i. e.*, 3.4 per cent., or 44 in every 1,000. It follows that 63.9 less in every 1,000 die now than in former years.—*Med. Rec.*

Death from Ether.—A death has occurred at Bellevue Hospital, the result of the inhalation of ether.—*Ibid.*

